# The TIM Barrel Architecture Facilitated the Early Evolution of Protein-Mediated Metabolism

**DOI:** 10.1007/s00239-015-9722-8

**Published:** 2016-01-05

**Authors:** Aaron David Goldman, Joshua T. Beatty, Laura F. Landweber

**Affiliations:** Department of Biology, Oberlin College, Oberlin, OH 44074 USA; Department of Ecology and Evolutionary Biology, Princeton University, Princeton, NJ 08544 USA

**Keywords:** TIM barrel, RNA–protein world, LUCA, RNA world, Prebiotic chemistry

## Abstract

The triosephosphate isomerase (TIM) barrel protein fold is a structurally repetitive architecture that is present in approximately 10 % of all enzymes. It is generally assumed that this ubiquity in modern proteomes reflects an essential historical role in early protein-mediated metabolism. Here, we provide quantitative and comparative analyses to support several hypotheses about the early importance of the TIM barrel architecture. An information theoretical analysis of protein structures supports the hypothesis that the TIM barrel architecture could arise more easily by duplication and recombination compared to other mixed α/β structures. We show that TIM barrel enzymes corresponding to the most taxonomically broad superfamilies also have the broadest range of functions, often aided by metal and nucleotide-derived cofactors that are thought to reflect an earlier stage of metabolic evolution. By comparison to other putatively ancient protein architectures, we find that the functional diversity of TIM barrel proteins cannot be explained simply by their antiquity. Instead, the breadth of TIM barrel functions can be explained, in part, by the incorporation of a broad range of cofactors, a trend that does not appear to be shared by proteins in general. These results support the hypothesis that the simple and functionally general TIM barrel architecture may have arisen early in the evolution of protein biosynthesis and provided an ideal scaffold to facilitate the metabolic transition from ribozymes, peptides, and geochemical catalysts to modern protein enzymes.

**Electronic supplementary material:**

The online version of this article (doi:10.1007/s00239-015-9722-8) contains supplementary material, which is available to authorized users.

## Introduction

**Fig. 1:**
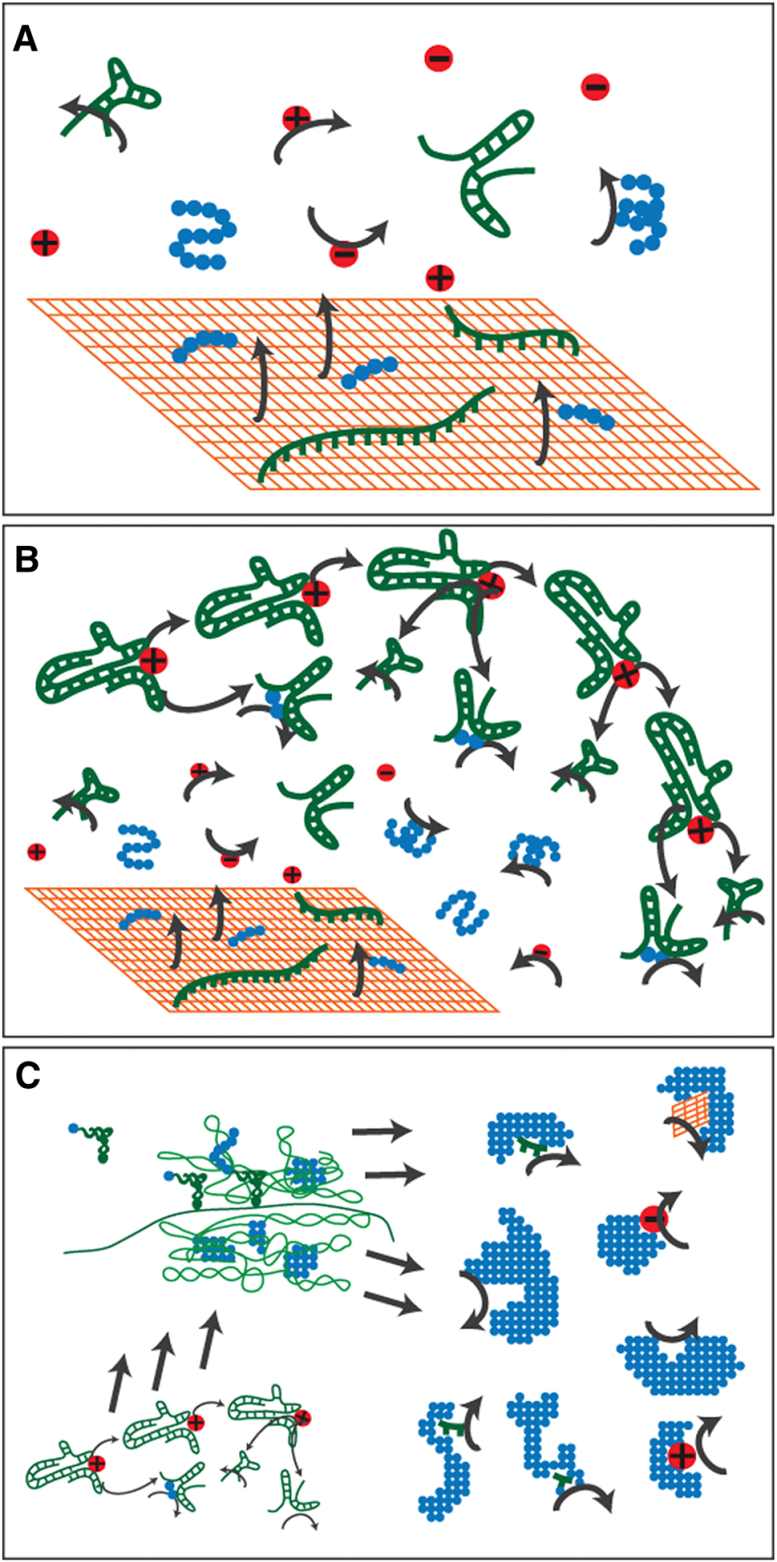
A well-supported scheme for the development of metabolism during the emergence of life. **a** Geochemical reactions catalyzed by mineral surfaces (*orange*) (Wächtershäuser 1988; Ertem and Ferris 1998) and metal ions (*red*) (Mulkidjanian and Galperin 2009) produce macromolecules such as amino acids (*blue*) and nucleotides (*green*) that polymerized into short peptides (Huber et al. 2003) and oligonucleotides (Huang and Ferris 2006) which, themselves, facilitated useful reactions. **b** A simple genetic system arose in which RNA genes encoded functional ribozymes (Gilbert 1986). This RNA-only genetic system was most likely dependent on the geochemical regime from which it emerged and may have co-evolved with catalytic peptides (Caetano-Anollés et al. 2007; Bowman et al. 2015). **c** Protein translation developed prior to the establishment of the DNA genome (Freeland et al. 1999), producing an RNA–protein system in which protein enzymes began to play a dominant role in metabolism. Modern enzyme cofactors derived from metals, nucleotides, and amino acids are thought to reflect the previous states in which reactions were catalyzed by ribozymes, peptides, metals, and minerals (White 1976; Szathmary and Maynard Smith 1994; Kyrpides and Ouzounis 1995; Wächtershäuser 1990; Yarus 1993; Mulkidjanian and Galperin 2009)

The emergence of life on Earth and the subsequent evolution of the last universal comment ancestor (LUCA) together represent a long process that passed through several distinct stages. Life emerged from an unknown geochemical context in which large proto-biomolecules were likely generated from smaller precursors. This prebiotic scenario must have been capable of producing precursor biopolymers that gave rise to the earliest genetic systems. The majority of evidence suggests that an RNA world scenario followed, in which a simple genetic system consisted of RNA genes that encoded a ribozyme-based metabolism (Gilbert 1986). While this hypothesis describes a predecessor to the current genetic system based on RNA or a similar biomolecule, it is possible, and some argue more likely, that many important metabolic reactions were catalyzed by amino acids, peptides, ions, and geochemical catalysts (Fig. 1b).

Additional evidence suggests that the system of biological protein synthesis by translation emerged from or co-evolved with this RNA-based genetic system (Freeland et al. 1999; Cech 2000) and that the establishment of the DNA genome followed (Forterre 2002; Goldman and Landweber 2012). By the time of LUCA, life seems to have evolved a sophisticated translation system (Goldman et al. 2010), a proteome consisting of a large percentage of modern protein architectures (Wang et al. 2007), extensive metabolic networks (Kyrpides et al. 1999; Goldman et al. 2012) and a stable, protein-rich, membrane that supported energy transduction via ATP synthase (Becerra et al. 2007; Lane et al. 2010), all encoded by a genome with hundreds or thousands of gene families (Ouzounis et al. 2006; Goldman et al. 2013). This biological complexity may have been primarily due to the transition from a metabolism based on ribozymes, peptides, and geochemical catalysts to a metabolism based on genetically encoded protein enzymes (Fig. 1c).

The first encoded proteins may have been simple, short peptides that acted as cofactors of ribozymes (Szathmary and Maynard Smith 1994). At later stages of ancient evolution, proteins may have become dependent on organic (Szathmary and Maynard Smith 1994; Kyrpides and Ouzounis 1995) and inorganic (Wächtershäuser 1990; Yarus 1993; Mulkidjanian and Galperin 2009) cofactors that reflect the ribozyme and abiotic catalysts that played important roles prior to the evolution of protein-mediated metabolism (White 1976).

Here, we examine whether the TIM barrel protein architecture could have played a fundamental role in this transition. TIM barrel structures (SCOP fold c.1; CATH topology 3.20.20) are generally composed of eight repeats of a β-strand and an α-helix, (β/α)_8_. The repetitive nature of this architecture led Gilbert and Glynias (1993) to propose that TIM barrel structures were originally encoded as single β/α units that were translated and subsequently formed active complexes in *trans*. More recent studies have found that TIM barrel proteins contain a number of homologous units within the overall structure (Frenkel and Trifonov 2005) and that some TIM barrel proteins appear to have evolved from the tandem duplication of quarter barrels (Richter et al. 2010) and half barrels (Lang et al. 2000; Henn-Sax et al. 2001), a process that can also be mimicked in vitro (Höcker et al. 2004).

TIM barrel proteins are perhaps most notable for their functional diversity. They are presently found in an astonishing 10 % of enzymes, usually as the catalytic domain (Copley and Bork 2000; Nagano et al. 2002). TIM barrel enzymes are known to catalyze at least five of the six major categories of enzyme functions as defined by the Enzyme Commission (Nagano et al. 2002) and have been assigned to the sixth category in the Uniprot (The Uniprot Consortium 2012) and KEGG (Kanehisa et al. 2012) databases. Copley and Bork (2000) found that 12 of the 23 TIM barrel superfamilies known at the time of their study appear to be evolutionarily homologous, but that functional similarity between these superfamilies is often not monophyletic, providing further support for the functional plasticity of the architecture. The process of novel gene evolution has recently been observed in TIM barrel proteins in real time and demonstrates the rapidity of acquiring new functions through a process of innovation followed by amplification and subsequent divergence (Näsvall et al. 2012). A recent success in the de novo design of a TIM barrel protein that does not share sequence similarity with those found in nature suggests that the potential functional range of the TIM barrel structure is greater than that represented in naturally occurring proteins (Huang et al. 2016).

The simple, repetitive structure of the TIM barrel, its functional diversity, and its broad use in a large number of protein enzymes and central metabolic pathways have led others to propose that the TIM barrel structure is both ancient (Gilbert and Glynias 1993; Yang et al. 2005; Wang et al. 2007) and played a central role in the early evolution of protein-mediated metabolism (Nagano et al. 2002; Anantharaman et al. 2003; Yamada and Bork 2009). In this study, we provide quantitative evidence that supports these hypotheses about the early emergence of TIM barrel superfamilies and their subsequent functional expansion in the context of early proteome evolution.

## Results and Discussion

### Evolvability of TIM Barrel Superfamilies

**Fig. 2:**
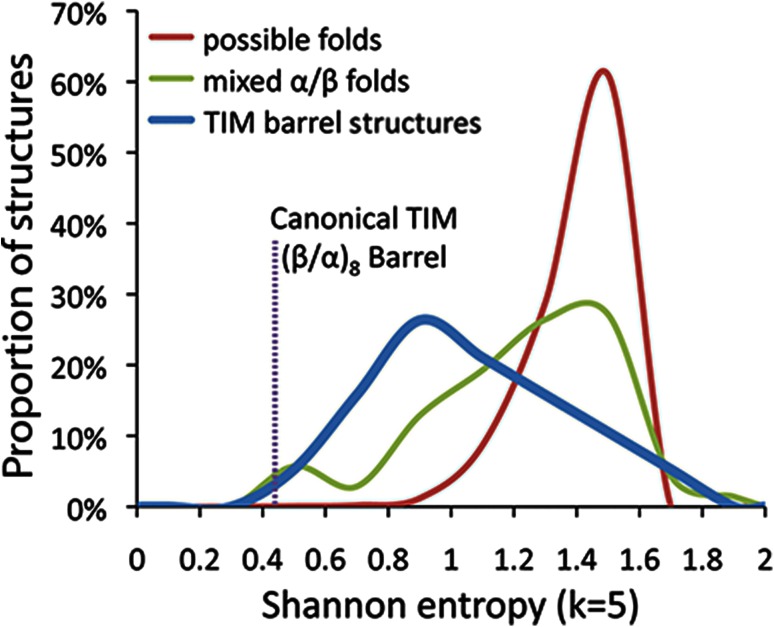
Comparison of the secondary-structural complexity of TIM barrels to other mixed α/β protein architectures. Structural repetitiveness is measured here as the linear Shannon entropy of secondary structure elements. Modern TIM barrel proteins exhibit a lower complexity (*i.e.*, more internal repetition) structure than most other mixed α/β structures. The canonical TIM barrel secondary structure (β/α)_8_ is far less complex than nearly all other mixed α/β structures. These results give quantitative support to the idea that TIM barrel proteins could have emerged easily from duplication and recombination of partial barrel structures during the early evolution of the protein repertoire (Richter et al. 2010; Lang et al. 2000; Henn-Sax et al. 2001)

The repetitiveness of the TIM barrel architecture has led a number of authors to conclude that the structure could have evolved from previously existing partial barrels through recombination and duplication (Straus and Gilbert 1985; Lang et al. 2000; Henn-Sax et al. 2001; Richter et al. 2010). To our knowledge, no study has yet compared the repetitiveness of the TIM barrel structure to other protein architectures with similar secondary structure populations. To do so, we measured the nonmetric Shannon entropy of the linear order of secondary structure elements along the protein chain (Fig. 2). Here, low Shannon entropy scores correspond to a simpler, more repetitive structure. The canonical TIM barrel pattern of (β/α)_8_ is very repetitive compared to all other possible combinations of alpha/beta secondary structure. Generally, secondary structure patterns from actual TIM barrel protein domains are not as repetitive as the canonical TIM barrel secondary structure pattern but are less complex than secondary structure patterns of other mixed α/β proteins.

The repetitiveness of the TIM barrel protein architecture suggests a higher likelihood that the structure can originate through common mechanisms of protein structural innovation, such as recombination or duplication. Previous work on simulated evolution of RNA sequences inferred that nucleotide sequence becomes less random over time through the formation of functional modules (Ancel and Fontana 2000). This trend was probably the case for early protein evolution as well, given the modular domain substructure of modern proteins (Chothia et al. 2003). At the level of secondary structure, however, the repetitive nature of the TIM barrel structure suggests that it could have easily been generated from smaller (β/α) proteins such as quarter and half barrels through some of the same processes responsible for exon shuffling and gene duplication. It is, therefore, not difficult to imagine that TIM barrel superfamilies could have easily, and perhaps repeatedly, arisen from smaller protein-coding genes during the early evolution of the protein repertoire.

### Taxonomic Breadth and Functional Range of TIM Barrel Superfamilies

**Fig. 3:**
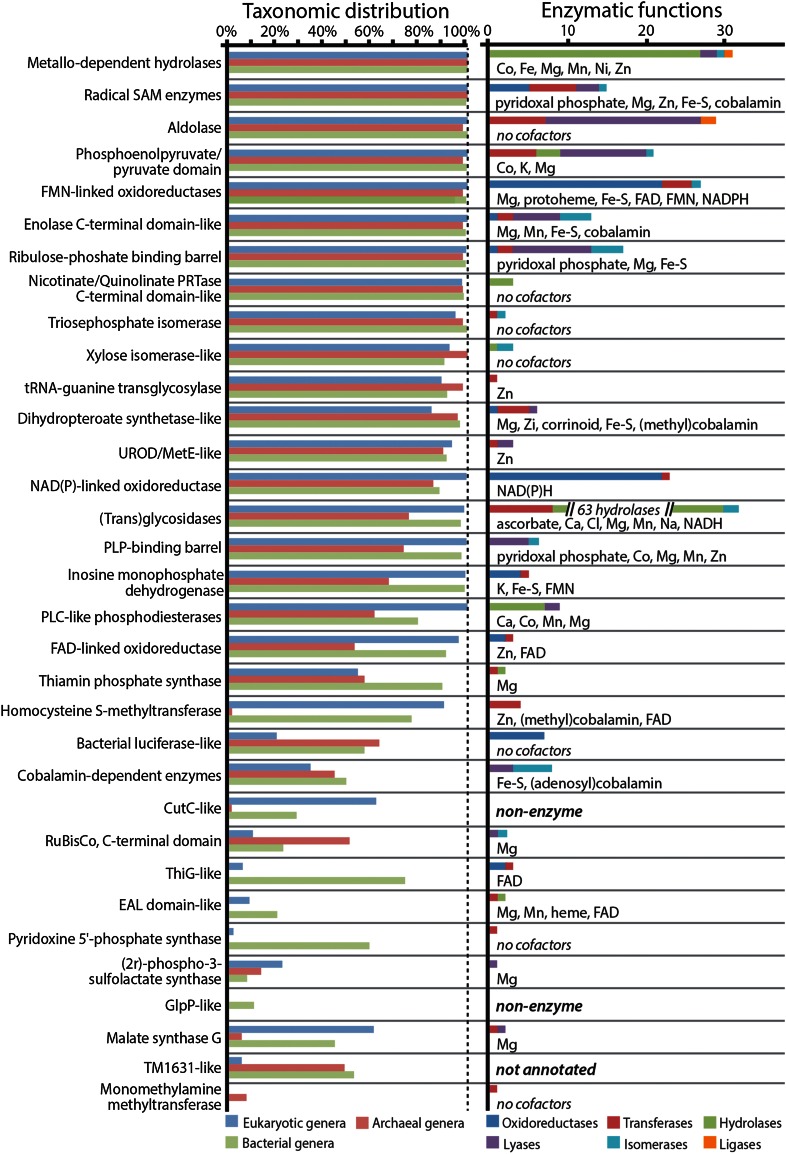
The taxonomic breadth and functional diversity of TIM barrel proteins. TIM barrel superfamilies as defined by the SCOP database are grouped by high structural similarity and low sequence similarity and are assumed to each be the result of a common ancestry. The percentage of genomes per taxonomic domain is presented for all 33 superfamilies. Most TIM barrel superfamilies are present in all three domains of life, indicating that they were also present at least as early as the last universal common ancestor (LUCA). These same taxonomically ubiquitous superfamilies show a very broad range of enzymatic functions (defined by the Enzyme Commission). The functional diversity of these putative TIM barrel superfamilies likely stems in part from the use of a wide range of metal-, nucleotide- and amino acid-derived cofactors, possibly reflecting their role in the transition to protein-mediated metabolism

As defined by the SCOP database (Andreeva et al. 2008), members of the same TIM barrel superfamily are related by a common origin. We evaluated the taxonomic distribution of TIM barrel superfamilies to approximate which superfamilies were likely present by the time of LUCA and whether a number of individual superfamilies exhibit a functional breadth comparable to the full range of TIM barrel proteins. Most of the 33 unique TIM barrel superfamilies have a broad taxonomic distribution, with 28 present in every domain of life and 20 present in the majority of genera within each of the three domains of life (Fig. 3, Online Resource 1).

We next assessed the functional breadth and cofactor usage of each TIM barrel family by gathering Enzyme Commission codes (Webb 1992) and cofactor usage data from annotations in the Uniprot database (The Uniprot Consortium 2012). Even though each superfamily presumably arose from a common ancestral protein, many of them have expanded to exhibit a broad functional range (Fig. 3). In the most extreme example, members of the (Trans)glycosylases superfamily are assigned 73 unique enzyme functions spanning three of the six major categories of enzyme function as defined by the Enzyme Commission system. Five TIM barrel superfamilies perform enzyme functions spanning four of the six major categories, four other superfamilies perform enzyme functions spanning three of the six major categories, and fourteen other superfamilies perform enzyme functions spanning two of the six major categories. Interestingly, the Uniprot database predicts two TIM barrel superfamilies to be capable of ligase function, which were previously thought to be outside the functional scope of TIM barrel enzymes (Nagano et al. 2002). A similar result is reported in the MANet database (Kim et al. 2006).

TIM barrel enzymes appear to achieve this functional breadth through the use of a broad range of metal cofactors, as well as cofactors derived from nucleotides and amino acids (The Uniprot Consortium 2012). Six TIM barrel superfamilies use iron-sulfur cofactors and seven use zinc cofactors. Both of these inorganic cofactors have been proposed to reflect mineral and ion catalysts important to ancient life (Wächtershäuser 1990; Mulkidjanian and Galperin 2009). Three TIM barrel superfamilies use the peptide-derived cofactors, corrinoid, heme, and protoheme, while twelve use nucleotide-derived cofactors, cobalamin, FAD, FMN, NADH, and NADPH. These cofactors have been proposed to reflect the transition from RNA-mediated metabolism to protein-mediated metabolism (Szathmary and Maynard Smith 1994; Kyrpides and Ouzounis 1995). A recent study by Caetano-Anollés et al. (2012) surveyed cofactor usage for the 54 protein fold families that they identify as being the most ancient. Cofactor usage among the three TIM barrel families in this survey was found to be limited to the flavin-related cofactors, which they identify as being among the most ancient.

### Comparison of the TIM Barrel Functional Range to Other Ancient Folds

**Fig. 4:**
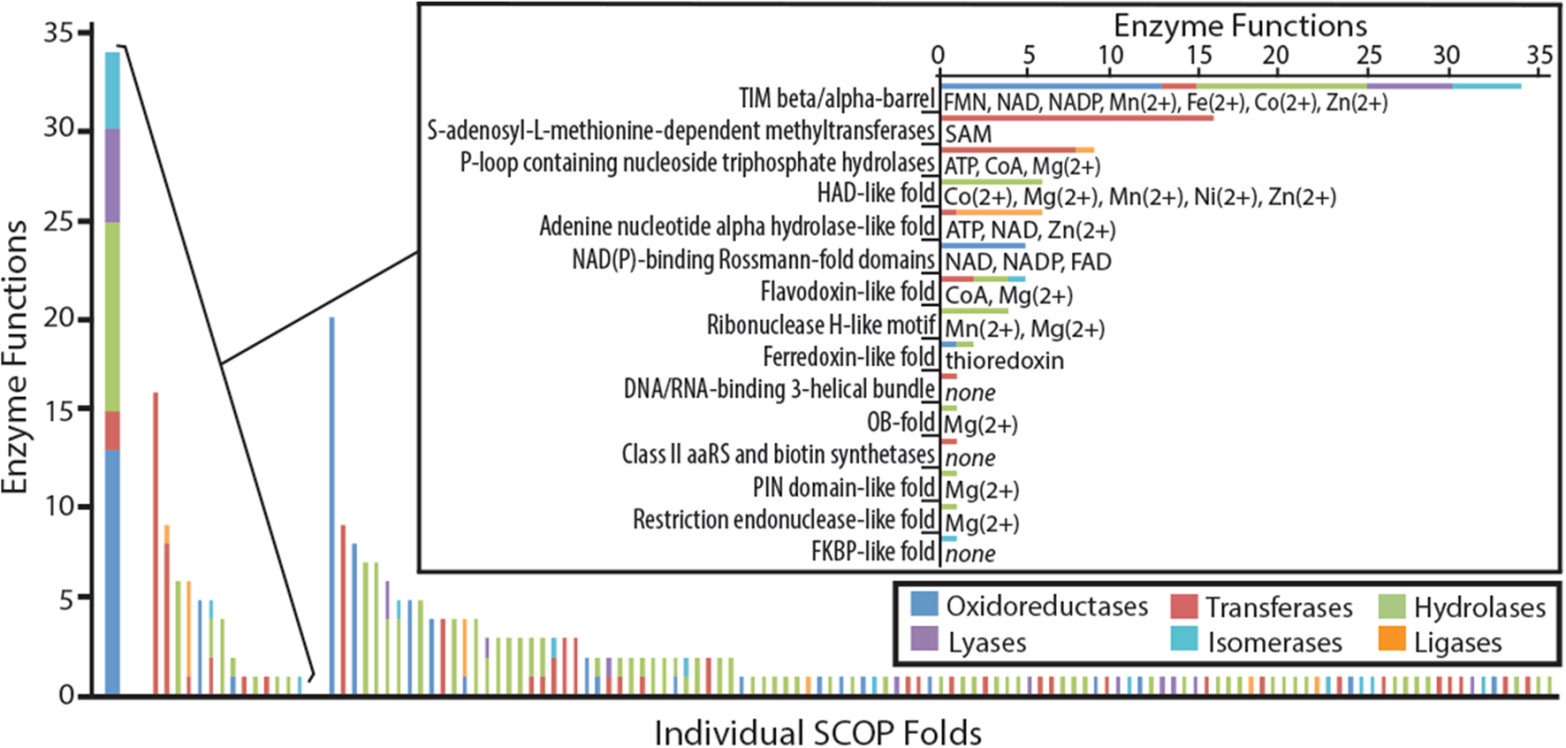
Comparison of the functional diversity of protein folds. The number of unique enzymatic functions performed by single-domain proteins of a given fold are presented as a histogram and color-coded by Enzyme Commission functional category. Ancient folds (Yang et al. 2005; Wang et al. 2007) are separated from the others in order to determine whether the breadth of the TIM barrel fold is, in part, due to its age. Single-domain TIM barrel proteins impart 34 unique functions spanning five major Enzyme Commission categories. This functional range is 70 % greater than the next most functionally broad structure. Single-domain TIM barrel proteins also use the broadest range of enzymatic cofactors, including the putatively ancient cofactors discussed in the main text

This and other studies have shown that the TIM barrel fold is functionally broad. We compare the breadth of TIM barrel functions to that of other folds. The TIM barrel domains of multidomain proteins are usually the catalytic domain (Nagano et al. 2002), but this is not the case for other folds, which may be present in multidomain enzymes but provide a structural or coenzymatic role. In order to avoid false attribution of functional annotations of protein folds due to their presence in multidomain proteins, we created a database consisting only of single-domain proteins and their functional annotations. Putatively ancient folds, defined by the overlap between predictions from Yang et al. (2005) and Wang et al. (2007), were compared separately in order to show that the breadth of TIM barrel functions is not due merely to its age (Fig. 4).

Single-domain TIM barrel proteins impart 13 unique oxidoreductase functions, 2 unique transferase functions, 10 unique hydrolase functions, 5 unique lyase functions, and 4 unique isomerase functions. This total of 34 unique functions is 70 % greater than the number of functions of the next most enzymatically broad fold, the Cytochrome P450 fold. The next four most functionally broad ancient folds after the Cytochrome P450 fold, are the S-adenosyl-l-methionine-dependent methyltransferases, P-loop containing nucleoside triphosphate hydrolases, UDP-glycosyltransferase/glycogen phosphorylase fold, and double-stranded beta-helix fold.

Only TIM barrel single-domain proteins are capable of five major categories of enzyme function. The flavodoxin-like fold is capable of performing enzyme functions from three different functional categories and all other single-domain proteins are only capable of one or two of the major categories of enzyme function. The comparison of the TIM barrel functional range to that of other protein folds suggest a propensity for broad enzymatic function and cofactor usage among other ancient proteins, but not to the extent of TIM barrel proteins.

### Relationship of TIM Barrel Functional Breadth to Cofactor Usage

Given the greater breadth of enzymatic function and cofactor usage exhibited by the TIM barrel structure, overall, and by individual TIM barrel superfamilies, we sought to investigate the relationship between functional range and cofactor usage in TIM barrel proteins. The correlation between cofactor usage and functional breadth for TIM barrel superfamilies compared to all protein superfamilies is shown in Table 1. The functional breadth of TIM barrel superfamilies strongly correlates with cofactor usage; however, the same analysis performed on all superfamilies shows a negligible relationship between functional breadth and cofactor usage.

**Table 1:** Correlations between cofactor usage and enzymatic breadth for TIM barrel superfamilies and superfamilies in general

	Variables	Correlation
TIM barrel superfamilies	Number of enzyme functions^a^ vs. number of cofactor	*r* = 0.56, *p* = 0.00078
	Number of enzyme categories^b^ vs. number of cofactor	*r* = 0.62, *p* = 0.00012
All superfamilies	Number of enzyme functions^a^ vs. number of cofactor	*r* = 0.16, *p* = 0.044
	Number of enzyme categories^b^ vs. number of cofactor	*r* = 0.0083, *p* = 0.92

^a^Enzyme functions are defined as complete Enzyme Commission codes

^b^Enzyme categories are defined as the first term of Enzyme Commission codes

Having established a distinctive relationship between functional breadth and cofactor usage in TIM barrel superfamilies, we performed a linear regression analysis to separate the contributions of cofactor usage and superfamily age to the function breadth of proteins in a superfamily. As a proxy for age, we used either the predicted presence in LUCA, defined as the presence in at least 80 % of genera in all three taxonomic domains, or the predicted absence in LUCA, defined by the presence in fewer than 25 % of genera in at least one domain of life.

The resulting linear regression formula fit the data with an *R*^2^ value of 0.63, indicating that cofactor usage and presence in LUCA together explain nearly two thirds of the variance in functional breadth within TIM barrel superfamilies. The relative weighting of cofactor usage was 2.81 (*p* = 0.0031), while the relative weighting of whether or not the superfamily was present in LUCA was 8.67 (*p* = 0.043). Although the functional breadth of a superfamily appears to depend considerably on its presence in LUCA, this analysis also shows that the range of cofactor usage is an important factor contributing to TIM barrel functional breadth independent of the age of the superfamily.

### Metabolic Analysis of TIM Barrel Functions

We also sought to better understand the metabolic distribution of functions performed by TIM barrel proteins generally and within individual superfamilies with a large functional range. The patchwork model of metabolic evolution predicts that metabolic pathways composed of multiple enzymes with specific functions evolved from a smaller number of functionally general enzymes that performed multiple catalytic functions (Yamada and Bork 2009). Recent evidence shows that TIM barrel proteins exhibit a pattern of distribution in modern metabolic pathways consistent of the patchwork model versus other models of metabolic pathway evolution (Caetano-Anollés et al. 2009).

To further test this hypothesis, we mapped the complete set of TIM barrel protein functions onto the KEGG global metabolic map (Figures S1 and S2). The complete set of TIM barrel functions exhibits a broad metabolic distribution across nearly all categories of enzymatic function. Next, we mapped the subset of functions performed by the three most functionally broad TIM barrel superfamilies, (Trans)glycosidases (c.1.8), metallo-dependent hydrolases (c.1.9), and aldolase (c.1.10). In all three cases, the metabolic distribution of functions within a single superfamily is mostly localized to adjacent pathways in the same metabolic category. (Trans)glycosidase superfamily functions appear most often in starch and sucrose metabolism (Figure S3), metallo-dependent hydrolase superfamily functions appear most often in nucleotide metabolism (Figure S4), and aldolase superfamily functions appear most often in sugar and energy metabolism (Figure S5).

In the case of (Trans)glycosidase superfamily functions and Aldolase superfamily functions, many members of the same superfamily occupy successive functions within a metabolic pathway. This localization of enzymatic functions performed by members of the same superfamily is consistent with the patchwork model of metabolic pathway evolution in which functional divergence or subfunctionalization within a protein family leads to functional specialization of enzymes within the metabolic pathway.

### The TIM Barrel Fold and the Early DNA Genome

The property of possessing a DNA genome most likely arose after protein enzymes capable of ribonucleotide reduction began to produce deoxyribonucleotides (Freeland et al. 1999). The anaerobic ribonucleotide reductase (class III) has been considered the oldest of these enzymes (Torrents et al. 2002; Nordlund and Reichard 2006) because it functions under anaerobic conditions similar to those under which most life would have evolved prior to the oxidation of the Earth’s crust, atmosphere, and ocean 2.4–2.7 billion years ago (Catling and Claire 2005). Anaerobic ribonucleotide reductase necessarily works as part of a two-component system along with a predicted (β/α)_4_ barrel enzyme, ribonucleotide reductase activating protein, which creates an initial glycyl radical (Nicolet and Drennan 2004). Lundin et al. (2015) recently argued that the class II radical-generating mechanism is likely older than the class III mechanism because the class II reaction does not require a separate enzyme. But those authors do leave open the possibility that the class III activating protein was coopted from another pathway, making its early evolution more likely.

**Fig. 5:**
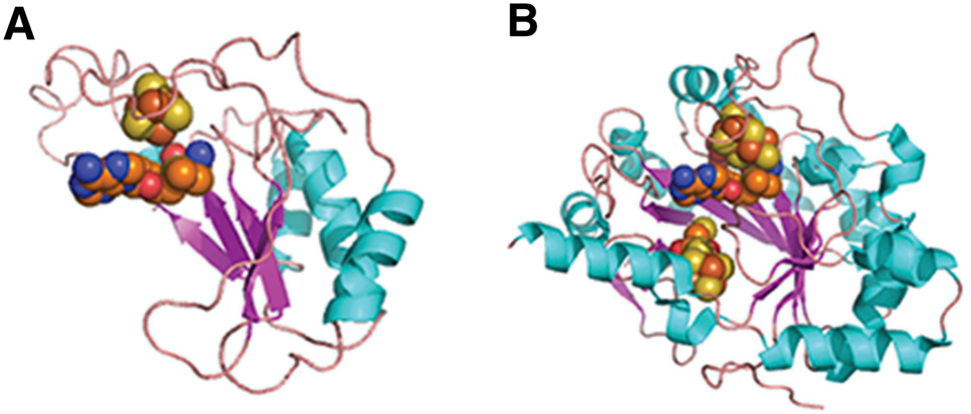
Structural similarity between the ribonucleotide reductase activating enzyme and a TIM barrel protein. The structure of ribonucleotide reductase activating enzyme, the radical redox component of potentially the earliest enzyme capable of making deoxyribonucleotides, is predicted to have a half TIM barrel structure (**a**) that most closely resembles the structure of the TIM barrel protein, 4Fe–4S-pyruvate formate-lyase activating enzyme (**b**), over any other structure in the Protein Data Bank. The iron-sulfur cluster and S-adenosyl methione cofactors used by ribonucleotide reductase activating enzyme were modeled based on superimposition with the 4Fe–4S-pyruvate formate-lyase activating enzyme

We find that ribonucleotide reductase activating protein is predicted by structural similarity to be a half TIM barrel (Fig. 5a) that most closely matches a component of the full TIM barrel protein, 4Fe–4S-pyruvate formate-lyase activating enzyme (Fig. 5b). Both proteins create a radical using supposedly ancient cofactors, iron-sulfur clusters, and S-adenosyl methionine. Structural similarity predicts that the ribonucleotide reductase activating protein is associated with the radical SAM enzymes superfamily (SCOP ID c.1.28) within the TIM barrel fold. If the early evolution of the class III RNR required the recruitment of a radical-generating enzyme from another pathway, it may have done so by exploiting the functional malleability of the TIM barrel structure.

## Conclusions

The modern form of protein-mediated metabolism emerged from a less capable system likely composed of ribozymes, peptide catalysts, and inorganic catalysts (Szathmary and Maynard Smith 1994; Lazcano and Miller 1999; Caetano-Anollés et al. 2007; Bowman et al. 2015). Previous studies have proposed that the inorganic cofactors, nucleotide-derived cofactors, and amino acid- or peptide-derived cofactors that facilitate modern protein catalysis may have ancient roots in this transition (Szathmary and Maynard Smith 1994; Wächtershäuser 1990; Yarus 1993; Mulkidjanian and Galperin 2009; White 1976). We have conducted a set of analyses focused on understanding the evolution of TIM barrel proteins in the context of early proteome evolution.

We showed that many TIM barrel superfamilies probably originated by the time of LUCA and that these superfamilies, themselves, demonstrate a high degree of catalytic diversity. Supporting our hypothesis, we find that inorganic cofactors and organic cofactors derived from nucleotides, amino acids, and peptides underlie this functional range in modern TIM barrel proteins and that this breadth of cofactor usage contributes to TIM barrel functional diversity in a manner unlike proteins, generally. It is not difficult to imagine that a significant functional expansion could have taken place prior to LUCA if TIM barrels had originated even earlier. Thus, the TIM barrel structure, with its evolutionarily malleable active site pocket, represents an ideal scaffold to facilitate the transition from ribozyme, peptide, and abiotic catalysts to modern protein-mediated metabolism.

## Methods

### Secondary Structure Complexity

Structural complexity was defined as the Shannon entropy of secondary structure elements along the protein chain from N-terminus to C-terminus. Real secondary structure data were acquired from the DSSP database (version 2.0: July, 2011) (Kabsch and Sander 1983). Domains corresponding to SCOP assignments were extracted from the PDB-formatted DSSP files and converted into strings of secondary structure assignments per residue. Secondary structure assignments of individual residues were then smoothed and converted to strings of alpha and beta elements. Shannon entropy was calculated on these strings, as well as the canonical TIM barrel string and all other possible mixed α/β strings of the same size (7–9 alpha helices and beta strands, respectively). Shannon entropy was calculated in a nonmetric form (that is, not normalized for length) using a substring length (k) of 5.

### Taxonomic Breadth and Functional Range of TIM Barrel Superfamilies

TIM barrel superfamilies are defined by SCOP version 1.75 (June, 2009) (Andreeva et al. 2008). The taxonomic survey of TIM barrel superfamilies was performed using the genome annotations in the superfamily database (Gough et al. 2001). We inverted the annotations of superfamilies per genome from the superfamily database to create a database of genomes per superfamily and removed genome redundancy at the genus level. Genomes were grouped into taxonomic domains and the percentage of total genomes per domain for each TIM barrel superfamily was calculated. The eukaryotic domain included genomes from 236 genera, the archaeal domain included genomes from 66 genera, and the bacterial domain included genomes from 515 genera.

Enzymatic functions of TIM barrel enzymes and their cofactor and coenzyme associations were determined by a survey of annotations from the Uniprot database release 2012_06. Enzymatic functions were defined as Enzyme Commission codes listed in the Uniprot annotation for individual proteins. The complete list of Enzyme Commission codes were collected for each superfamily and made nonredundant. Cofactors were annotated for individual proteins using the “Cofactor” designation within uniprot annotations. Lists of both enzyme commission codes and cofactors were assembled for each superfamily and made nonredundant.

### Comparison of the Functional Ranges of Folds

The database of single-domain proteins (Online Resource 2) was constructed by a series of database mapping steps. First, protein structures from the PDB with only one domain were identified using the SCOP database. Some of these PDB entries represent multidomain proteins for which only a fragment was solved due to the constraints of crystallization. These were removed from the database of single-domain proteins by identifying the corresponding Uniprot entry and comparing its sequence with the amino acid sequence of the protein structure. Proteins with a “subunit” entry in the Uniprot database were also removed to ensure that single-domain proteins were not members of larger multiprotein complex. Enzymatic functions and cofactor usage were identified from Uniprot annotations.

### Analysis of Cofactor Usage and Functional Breadth

All statistical analyses were performed using the StatPlus software package. In order to perform the linear regression analysis of the contributions of the cofactor breadth and the presence in LUCA, we separated TIM barrel proteins into a LUCA and non-LUCA set with the former defined by presence in at least 80 % of genera in all three taxonomic domains and the latter defined by presence in less than 25 % of genera in at least one domain of life. The LUCA set contained superfamilies, c.1.9, c.1.28, c.1.10, c.1.12, c.1.4, c.1.11, c.1.2, c.1.17, c.1.1, c.1.15, c.1.20, c.1.21, c.1.22, c.1.7, and c.1.8. The non-LUCA set contained superfamilies, c.1.26, c.1.16, c.1.19, c.1.30, c.1.14, c.1.31, c.1.33, c.1.24, c.1.27, c.1.29, c.1.13, c.1.32, and c.1.25. The superfamilies, c.1.6, c.1.5, c.1.18, c.1.23, and c.1.3, were excluded from the analysis because they did not fit either criteria.

### Metabolic Mapping of TIM Barrel Functions

TIM barrel enzyme functions were defined as Enzyme Commission codes and mapped onto the KEGG global metabolism (map 1100) using the KEGG webserver’s “user data mapping” function (Kanehisa et al. 2012).

### Analysis of Ribonucleotide Reductase Activating Protein

The structure of ribonucleotide reductase activating protein was predicted by submitting the NrdG sequence from *E. coli* to the I-TASSER webserver (Zhang 2008; Roy et al. 2010). I-TASSER identified 4Fe–4S-pyruvate formate-lyase activating enzyme as the closest structural homolog in the Protein Data Bank (Berman et al. 2000) through TM-score (Zhang and Skolnick 2005). The iron-sulfur cluster and S-adenosyl methionine cofactors of ribonucleotide reductase activating protein were modeled by alignment to the 4Fe–4S-pyruvate formate-lyase activating enzyme X-ray diffraction structure (3C8F) using the PyMol molecular viewer (DeLano 2008).

## Electronic supplementary material

Supplementary material 1 (TXT 5 kb)

Supplementary material 2 (TXT 18 kb)

Supplementary material 3 (TIFF 2145 kb)

Supplementary material 4 (TIFF 2145 kb)

Supplementary material 5 (TIFF 2145 kb)

Supplementary material 6 (TIFF 2144 kb)

Supplementary material 7 (TIFF 2145 kb)

Supplementary material 8 (PDF 57 kb)
