# Supplementary figures and images for: The TIM Barrel Architecture Facilitated the Early Evolution of Protein-Mediated Metabolism
Source: J Mol Evol. 2016 Jan 5;82:17–26. doi: 10.1007/s00239-015-9722-8 (PMC4709378; doi:10.1007/s00239-015-9722-8)

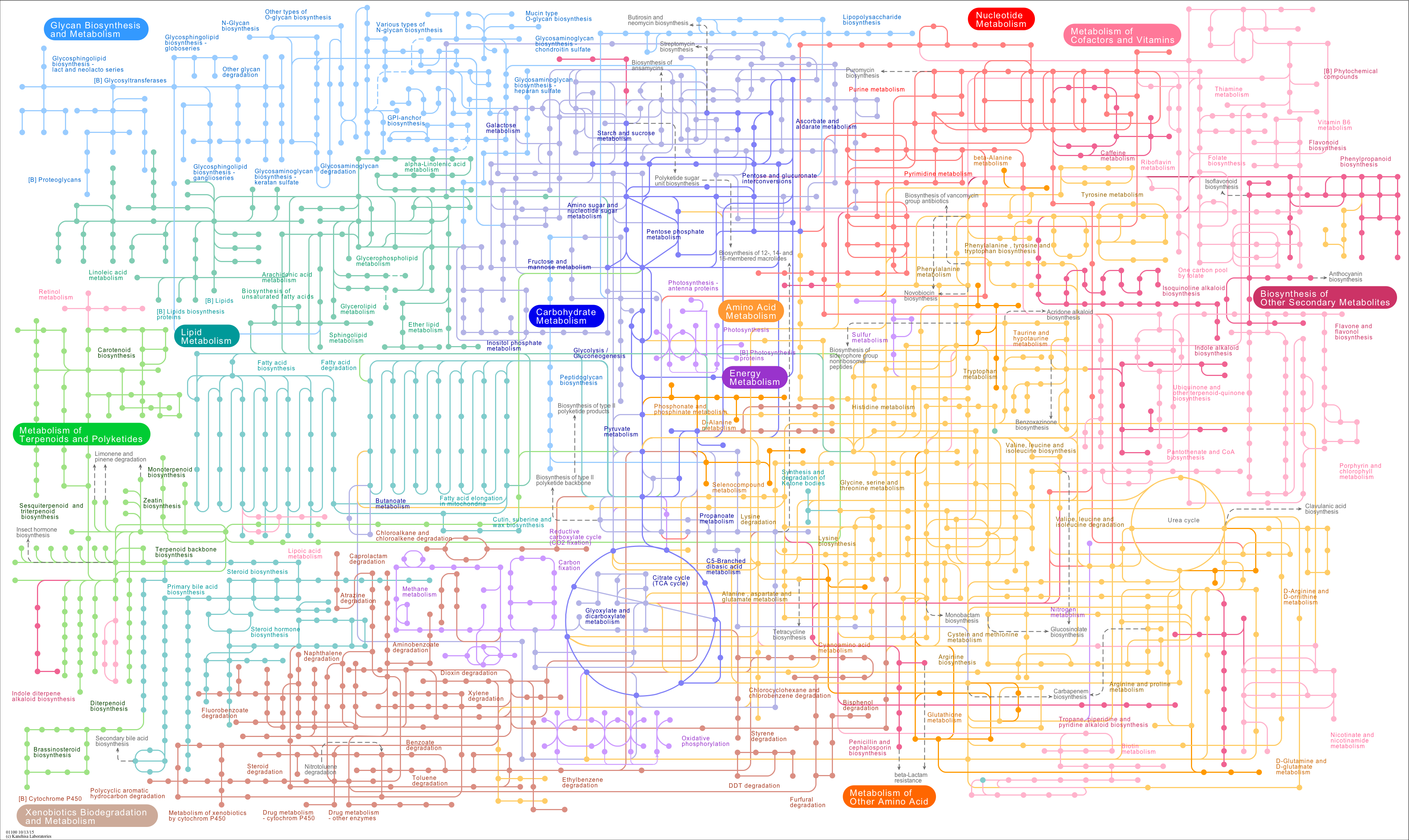

Supplement: Supplementary file 3 — Supplementary material 3 (TIFF 2145 kb) [file 239_2015_9722_MOESM3_ESM.tif]

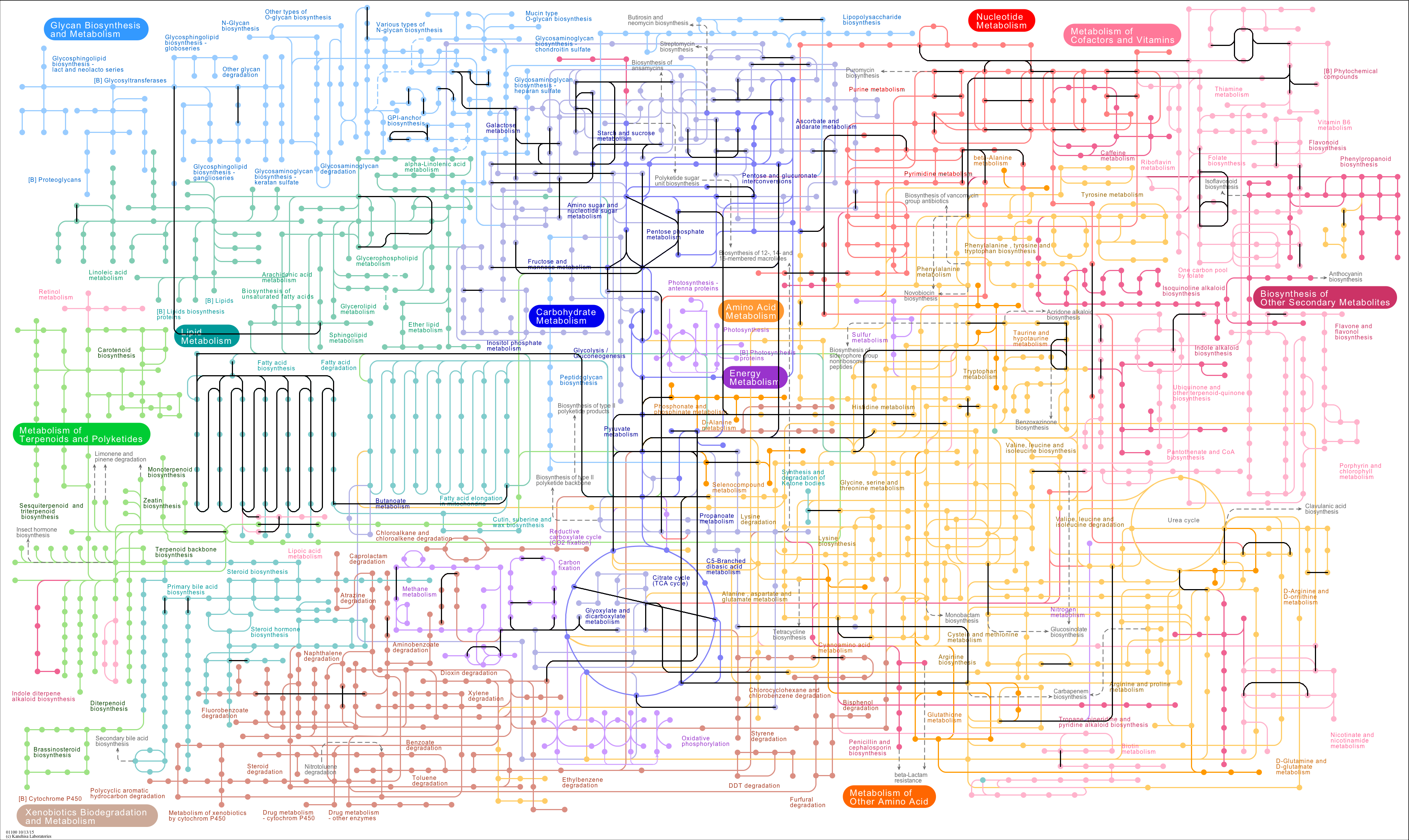

Supplement: Supplementary file 4 — Supplementary material 4 (TIFF 2145 kb) [file 239_2015_9722_MOESM4_ESM.tif]

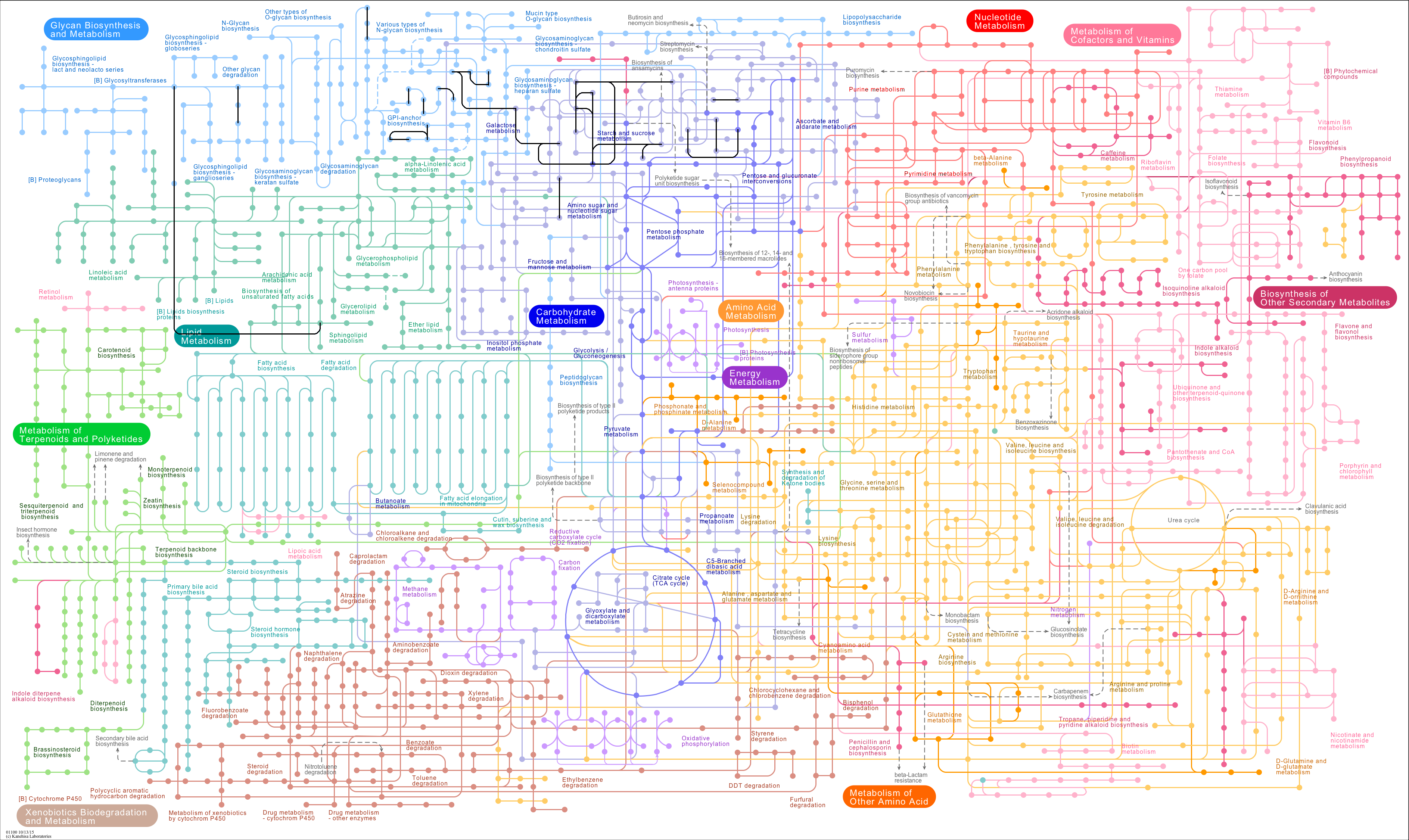

Supplement: Supplementary file 5 — Supplementary material 5 (TIFF 2145 kb) [file 239_2015_9722_MOESM5_ESM.tif]

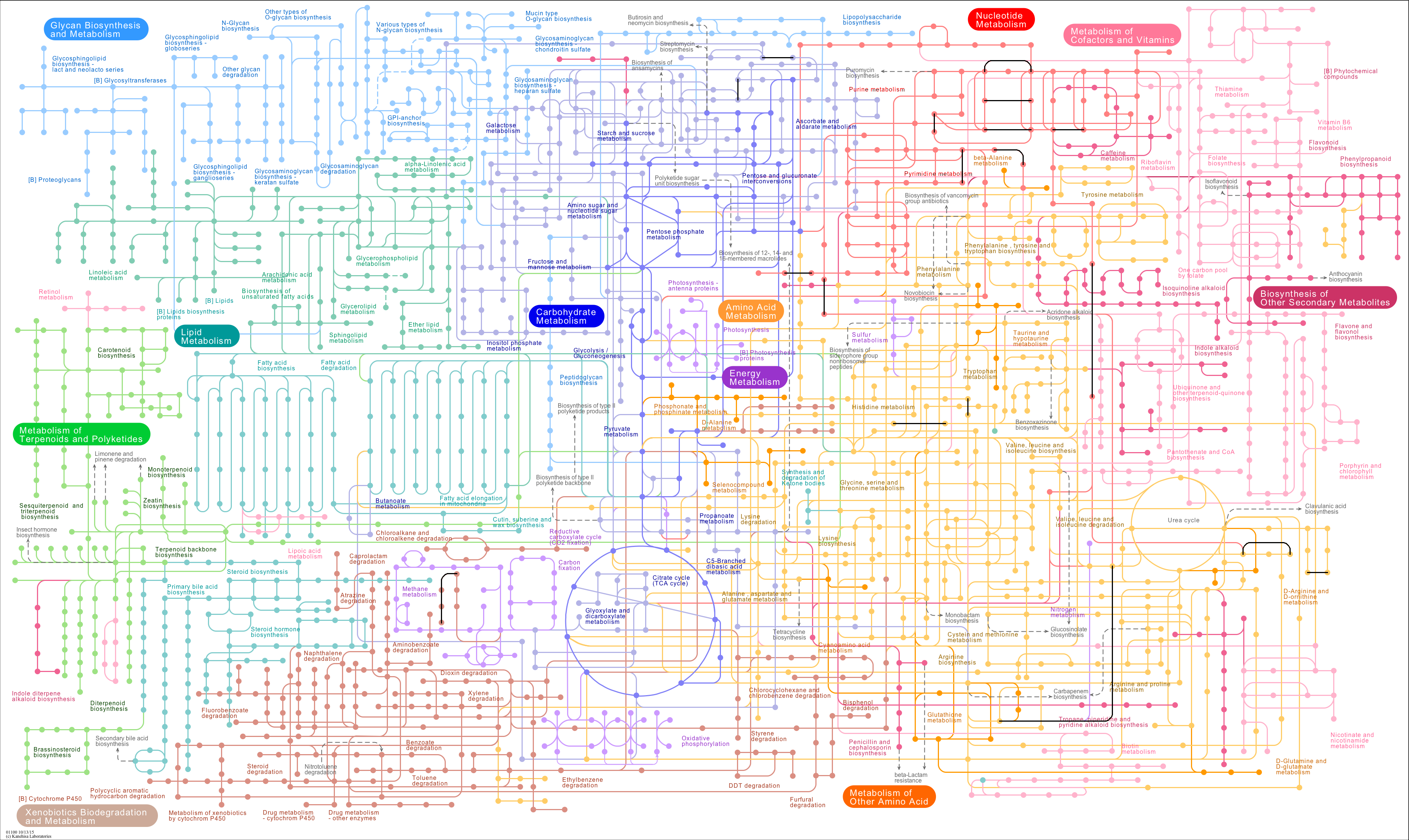

Supplement: Supplementary file 6 — Supplementary material 6 (TIFF 2144 kb) [file 239_2015_9722_MOESM6_ESM.tif]

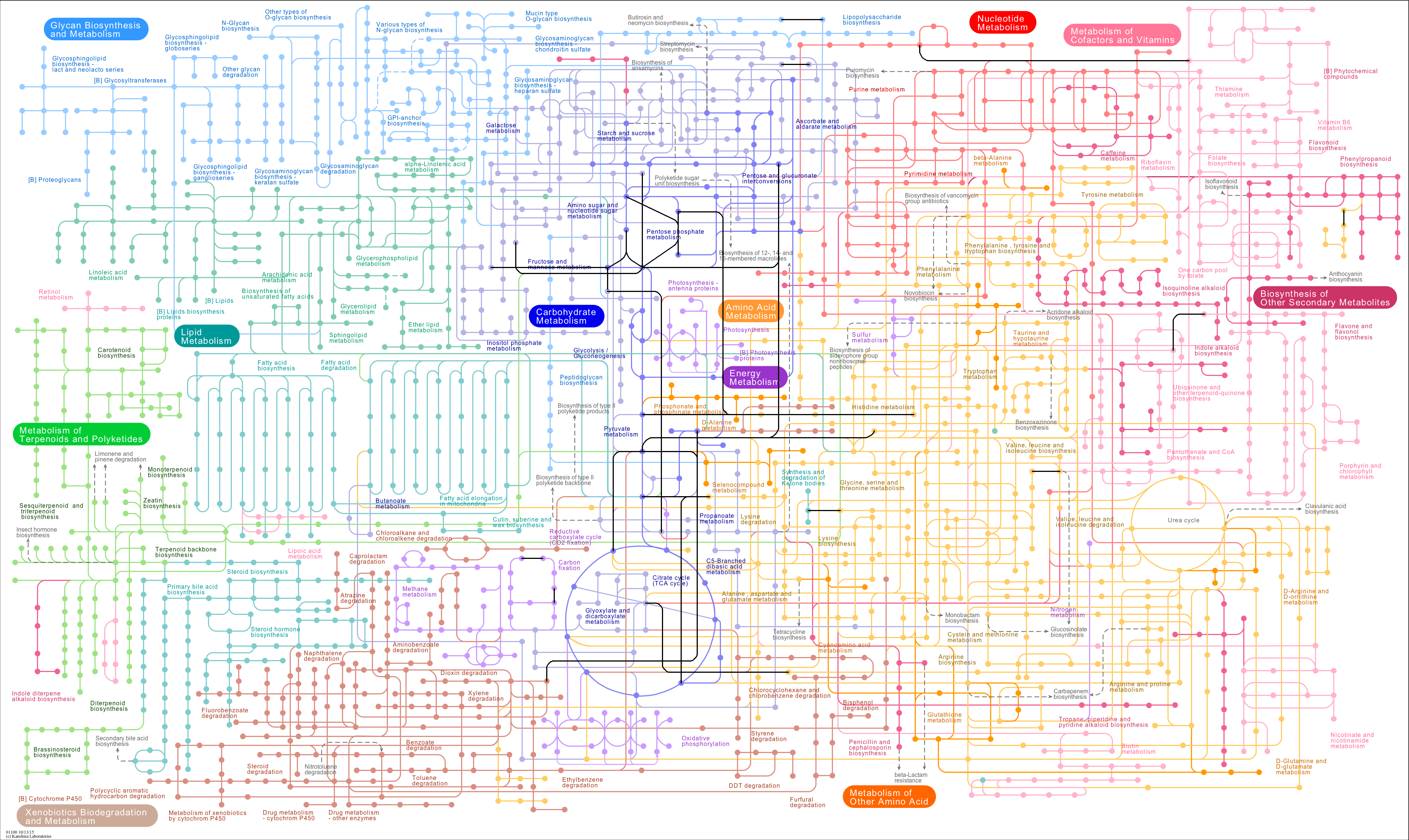

Supplement: Supplementary file 7 — Supplementary material 7 (TIFF 2145 kb) [file 239_2015_9722_MOESM7_ESM.tif]
