# Supplementary material for: The TIM Barrel Architecture Facilitated the Early Evolution of Protein-Mediated Metabolism
Source: J Mol Evol. 2016 Jan 5;82:17–26. doi: 10.1007/s00239-015-9722-8 (PMC4709378; doi:10.1007/s00239-015-9722-8)
Supplement: Supplementary file 8 — Supplementary material 8 (PDF 57 kb) [file 239_2015_9722_MOESM8_ESM.pdf]

Title: The TIM Barrel Architecture Facilitated the Early Evolution of Protein-Mediated Metabolism

Journal: Journal of Molecular Evolution

Authors: Aaron David Goldman*, Joshua T. Beatty, Laura F. Landweber

*agoldman@oberlin.edu

Supplemental Figures Legends:

Figure S1. The KEGG global metabolism map (map 1100).

Figure S2. The KEGG global metabolism with TIM barrel protein functions mapped onto it (shown as black lines connecting metabolites). Functions are defined by Enzyme Commission codes and mapped onto the KEGG global metabolism map using the KEGG webserver's "user data mapping" function. TIM barrel protein functions span a broad range of metabolic pathways.

Figure S3. The KEGG global metabolism with all (Trans)glycosidase superfamily functions mapped onto it (shown as black lines connecting metabolites). Functions are defined by Enzyme Commission codes and mapped onto the KEGG global metabolism map using the KEGG webserver's "user data mapping" function. (Trans)glycosidase superfamily functions appear most often in starch and sucrose metabolism. Members of the same superfamily occupy successive functions in the metabolic pathway, suggesting a history predicted by the patchwork model of metabolic pathway evolution.

Figure S4. The KEGG global metabolism with Metallo-dependent hydrolase superfamily functions mapped onto it (shown as black lines connecting metabolites). Functions are defined by Enzyme Commission codes and mapped onto the KEGG global metabolism map using the KEGG webserver's "user data mapping" function. Metallo-dependent hydrolase superfamily functions appear most often in nucleotide metabolism.

Figure S5. The KEGG global metabolism with Aldolase superfamily functions mapped onto it (shown as black lines connecting metabolites). Functions are defined by Enzyme Commission codes and mapped onto the KEGG global metabolism map using the KEGG webserver's "user data mapping" function. Aldolase superfamily functions appear most often in sugar and energy metabolism. Members of the same superfamily occupy successive functions in the metabolic pathway, suggesting a history predicted by the patchwork model of metabolic pathway evolution.
